# Editorial: New drugs, approaches and strategies for multiple sclerosis treatment

**DOI:** 10.3389/fnins.2024.1372140

**Published:** 2024-01-30

**Authors:** Rafael Coveñas, Pilar Marcos, Arturo Mangas

**Affiliations:** ^1^Laboratory of Neuroanatomy of the Peptidergic Systems, Institute of Neurosciences of Castilla and León (INCYL), University of Salamanca, Salamanca, Spain; ^2^Human Neuroanatomy Laboratory, Faculty of Medicine, Regional Centre of Biomedical Research (CRIB), University of Castilla-La Mancha, Albacete, Spain

**Keywords:** multiple sclerosis, magnetic resonance imaging (MRI), experimental autoimmune encephalomyelitis, arginine vasopressin hormone (AVP) receptor, remyelination

Multiple sclerosis (MS), twice more common in women than in men and whose incidence is growing worlwide, is a chronic inflammatory, autoimmune, and neurodegenerative disease which courses over several decades. It is characterized by demyelination, axonal degeneration, and neuronal death which main targets are axons, myelin and oligodendrocytes (Radandish et al., 2021). The disease, in which immune cells are mainly involved, shows a complex evolution and different ways of manifestation in the patients because it depends on the region of the central nervous system affected. MS shows a different geographic distribution (with a North-South gradient in the Northern hemisphere) and is generally diagnosed in individuals aged from 20 to 45 years old. About 85% of the MS patients start with the relapsing-remitting form and after 12–15 years suffering from the latter form, the disease enters in the secondary progressive phase, being unfortunately the worsening continuous. Currently, most of the MS therapeutic strategies are directed against the inflammatory phase of the disease, important but not exclusive (Geffard et al., 2017). In fact, nerve disfunction and demyelination become more crucial processes in the progressive phase than the inflammatory mechanisms. Approved drugs for MS treatment do not change the course of the disease and to date, the most important challenge is the developing of new therapeutic strategies against this multifactorial disease (Gonzalez-Lorenzo et al., 2024). For this reason, new diagnostic tools and drugs must be urgently developed to efficiently fight the disease and to reduce the deleterious important side-effects promoted by the MS current treatments (e.g., cancer, leukopenia, hepatotoxicity), increasing the quality of life and decreasing the sequelae of patients suffering from MS (Mercadante, 2024).

An important aim of this Research Topic is to present the latest advances in MS diagnostic tools and therapies; the researchers participating have fully achieved this goal contributing to increase the knowledge on the disease. They have focused their research on the following issues: (1) The use of arginine vasopressin hormone (AVP) receptor antagonists in experimental autoimmune encephalomyelitis rodent models (Calvillo-Robledo et al.); (2) Remyelination, after administration of *Acer truncatum* oil, in a murine model of MS (Xue et al.); (3) A case report of a patient suffering from MS with imaging features of glymphatic failure benefitted from cerebrospinal fluid flow shunting (Scollato et al.); and (4) The signal intensity variations (SIVs) of MS lesions on magnetic resonance imaging (MRI) as a biomarker for the disability of MS patients (Sedaghat et al.). New diagnostic methods (e.g., PET scan, MRI) are essential tools for MS diagnosis, MS progression prediction, anticipation of new episodes, or worsening, and ultimately for a better MS understanding. In this sense, Sedaghat et al. from the University of San Diego (USA) have suggested that the SIVs of MS lesions on direct myelin imaging and standard clinical sequences could serve as an MRI biomarker for the disability of patients suffering from MS. They found that the higher the Expanded Disability Status Scale (EDSS) values, the more SIV of MS lesions. This important observation must be tested in a greater number of MS patients, but if confirmed, this promising finding could be used as a new MRI biomarker for the disability of MS patients. A second example of the importance of the diagnostic methods (MRI) has been presented by Scollato et al. from the University of Florence (Italy). They have presented a case report in which a patient with primary progressive MS and dilatation of the perivascular spaces [impairment of the glymphatic-lymphatic system (GLS)] had a transient improvement after cerebrospinal fluid shunt diversions. Although the processes involved in this improvement are currently unknown, this finding opens the door to study the disturbances of the cerebrospinal fluid on the GLS failure occurring in neurodegenerative processes. Calvillo-Robledo et al. from the Universidad Autónoma de Aguascalientes (Mexico) have reviewed the effects on the immune response mediated by conivaptan (an inhibitor of AVP receptor types 1 and 2); the administration of this inhibitor reduced the harmful effects promoted by the current therapies used in clinical practice. The authors concluded that conivaptan could be a promising therapeutic agent against MS accordingly to the results obtained in murine experimental autoimmune encephalomyelitis models. Finally, Xue et al. from the Xi'an Medical University (China) assume that the diet plays an important role in both MS course and pathogenesis. In this sense, they state that the oil administration of *Acer truncatum* is a promising treatment against some neurodegenerative diseases (Xue et al.). Accordingly, they studied remyelination processes after administration of the mentioned oil in a mouse model of demyelination induced by cuprizone, and they reported that this treatment counteracted demyelination. The level of myelin basic protein and the number of mature oligodendrocytes were increased in the demyelinated areas after treatment with *Acer truncatum* oil, and hence myelin repair was greatly enhanced in these areas. Taken together, the findings reported in this Research Topic in experimental animal models of MS as well as in patients suffering from MS open new promising research lines and therapeutic possibilities to improve MS diagnosis/treatment and to counteract the harmful side-effects promoted by current clinical MS treatments. Importantly, the decrease of side-effects could allow a long-term MS treatment with a determined drug.

Unfortunately, MS has no cure and only partial effective therapies are currently available; at most, these therapies only delay the course of the disease. In addition, they are mainly directed against the MS relapsing-remitting phases but not against the progressive phase of the disease. This means that the therapeutic strategy against MS must be focused, not only on inflammatory mechanisms, but also against other processes such as neuronal death, demyelination and oxidative stress (Geffard et al., 2017). Thus, drug candidates/combination of drugs for the treatment of MS must accomplish the following three requirements: (1) They must exert a global beneficial effect counteracting all the pathological mechanisms reported in the disease (e.g., inflammation, demyelination, neuronal death); (2) They must act against all the MS phases; and (3) They should produce no or minimal side-effects. This is the way to follow. This Research Topic advises future research lines with diagnostic and therapeutic potential that must be developed because novel possibilities for translational research are arising from the findings reported.

## Author contributions

RC: Writing—original draft, Writing—review & editing. PM: Writing—original draft, Writing—review & editing. AM: Writing—original draft, Writing – review & editing.

## References

[B1] Geffard M. Mangas A. Coveñas R. (2017). Follow-up of multiple sclerosis patients treated with Endotherapia. Biomed. Rep. 6, 307–313. 10.3892/br.2017.857 28451391 PMC5403692

[B2] Gonzalez-Lorenzo M. Ridley B. Minozzi S. del Giovane C. Perver G. Piggott T.. (2024). Immunomodulators and immunosuppresants for relapsing-remitting multiple sclerosis: a network meta-analysis. Cochrane Datab. Syst. Rev. 1, CD011381. 10.1002/14651858.CD011381.pub3 PMC10765473 38174776

[B3] Mercadante S. (2024). Palliative care aspects in multiple sclerosis. J. Pain Symptom Manage. 12, S0885–3924. 10.1016/j.jpainsymman.2024.01.006 38219965

[B4] Radandish M. Khalilian P. Esmaeil N. (2021). The role of distinct subsets of macrophages in the pathogenesis of MS and the impact of different therapeutic agents on these populations. Front. Immunol. 12, 667705. 10.3389/fimmu.2021.667705 34489926 PMC8417824

